# High accuracy, fiducial marker-based image registration of correlative microscopy images

**DOI:** 10.1038/s41598-019-40098-4

**Published:** 2019-03-01

**Authors:** Sajjad Mohammadian, Jantina Fokkema, Alexandra V. Agronskaia, Nalan Liv, Cecilia de Heus, Elly van Donselaar, Gerhard A. Blab, Judith Klumperman, Hans C. Gerritsen

**Affiliations:** 1 Molecular Biophysics, Debye Institute for Nanomaterials Science, Utrecht University, Utrecht, The Netherlands; 2 Section Cell Biology, Center for Molecular Medicine, University Medical Center Utrecht, Utrecht, The Netherlands

## Abstract

Fluorescence microscopy (FM) and electron microscopy (EM) are complementary techniques. FM affords examination of large fields of view and identifying regions of interest but has a low resolution. EM exhibits excellent resolution over a limited field of view. The combination of these two techniques, correlative microscopy, received considerable interest in the past years and has proven its potential in biology and material science. Accurate correlation of FM and EM images is, however, challenging due to the differences in contrast mechanism, size of field of view and resolution. We report an accurate, fast and robust method to correlate FM and EM images using low densities of fiducial markers. Here, 120 nm diameter fiducial markers consisting of fluorescently labelled silica coated gold nanoparticles are used. The method relies on recording FM, low magnification EM and high magnification EM images. Two linear transformation matrices are constructed, FM to low magnification EM and low magnification EM to high magnification EM. Combination of these matrices results in a high accuracy transformation of FM to high magnification EM coordinates. The method was tested using two different transmission electron microscopes and different Tokuyasu and Lowicryl sections. The overall accuracy of the correlation method is high, 5–30 nm.

## Introduction

Correlative light and electron microscopy (CLEM) combines the high sensitivity and large field of view of fluorescence microscopy with the high resolution of electron microscopy. Here, FM is used to rapidly visualize, localize and track fluorescent molecules in cells. This enables identification of regions of interest and can provide molecular information with high sensitivity. The EM images provide complementary, high resolution ultrastructural information. CLEM combines the power of both techniques and opens up the possibility to visualize rare (transient) events in cells or specific cells within complex tissues.

The contrast in FM and EM images, however, is very different in nature. In FM, only parts of the specimen that are fluorescently labelled with e.g. a fluorescent antibody are visible while in EM the contrast relies on electron density variations usually generated by heavy metal staining and visible throughout the specimen. This results in similar features having very different appearances in the FM and EM images. As a result, precise registration of the FM and EM images can be challenging.

Several approaches are employed to register FM to high magnification EM images. A straight-forward method is to manually identify and overlay regions in FM and high magnification EM images^1–5^. This method is strongly user dependent and its accuracy cannot be determined.

Another method is to identify features in FM that can be overlaid with the corresponding feature in low magnification TEM. Next, the low magnification EM image is correlated with the high magnification TEM image^6,7^. This method relies on (auto)-fluorescence in an ultra-thin section of the sample^7^ or the signal from Uranyl acetate under cryo conditions^6^. However, the applicability of this method is limited, as the 50–70 nm thin TEM sections give only a very low auto-fluorescence signal and cryo-fluorescence microscopy is rarely done on plastic embedded samples.

Fiducial markers that are visible in both EM and FM have proven to be very successful in correlating FM with EM images^8–10^. The drawback of this method is that for high precision image correlation in every EM image at least 6 fiducial markers must be present. This imposes limitations on the density of the fiducial markers. The maximum density is determined by the resolution of the fluorescence microscope, and as a result the minimum dimensions of the EM images should be on the order of several μm. Moreover, the high brightness of the fiducial markers may overwhelm the signal of for instance fluorescent antibodies and parts of the structures in the EM image may be covered by the markers. One way to overcome this is to use two types of fiducial markers; primary fiducials to correlate FM and low magnification EM images and secondary fiducials for low and high magnification EM images. In literature quantum dots and gold particles have been used as secondary fiducials^11^, but their use has been limited to regions only with enough fiducials.

This paper describes a new high accuracy correlation method. In contrast to the methods mentioned above no fiducials need to be present in the high magnification TEM image; only a low density of fiducial markers is required. Moreover, besides adding the fiducials no special sample treatment is required. Overlaying FM and high magnification EM images is divided into two steps; first the FM and low magnification EM images are correlated and next the low magnification EM image is correlated with the high magnification EM image. The FM to low magnification EM correlation is done using fluorescently labelled silica coated gold nanoparticles. These fiducials are easy to detect in FM and their gold core can be well discerned in EM, even at low magnifications. Low magnification EM and high magnification EM images are then correlated using cross correlation. EM images of different magnification can be easily correlated using cross correlation methods due to the presence of the same structures and the same contrast. Correlation of FM and high magnification EM is achieved by multiplying the two transformation matrices associated with the two correlation steps. The correlation accuracy is analyzed for each correlation step independently and also for the total transformation.

The method is demonstrated in different TEMs and using different samples, including Tokuyasu sections of HeLa Cells expressing lysosomal-associated membrane protein 1 conjugated to green fluorescent protein (LAMP1-GFP), HeLa Cells transiently transfected with mitochondrial import receptor subunit TOM20, immortalized FSHD myotubes stained for actin with Phalloidin-Alexa Fluor® 647 and Lowicryl sections of muscle biopsy from a FSHD patient labelled for Caveolin and C2C12 cells transiently transfected with hDUX4 and labelled for hDUX4. In some of the examples use is being made of both fluorescence immuno-labeling and immuno-gold labeling. The effect of sample shrinkage due to exposure to the electron beam is discussed and strategies to minimize shrinkage effects are provided.

## Materials and Methods

### Sample preparation

#### Preparation of TEM Grids with Fiducial Markers

15 nm diameter gold particles were synthesized^12^ and coated with a rhodamine B (Sigma Aldrich) labelled silica shell to obtain 120 nm diameter fluorescent gold/silica particles with concentration of 500 µgr/l. The silica coating and fluorophore incorporation was based on earlier work^13–18^ and was optimized to obtain monodisperse, bright and (photo)stable particles.

200 mesh hexagonal copper grids (Stork-Veco b.v.) were coated with Formvar film (Agar Scientific) and next with carbon. The carbon coated side was glow discharged for 15 s at a current of 10 mA (Cressington 208carbon); this surface treatment ensures homogenous dispersion of the fiducial markers. The stock solution of the fiducial markers in ethanol was diluted 1:250 in Milli-Q water and sonicated for 15 minutes (Branson 1200). Next, the glow discharged carbon coated sides of the grids were incubated for 10 min by placing the grids on 25 µl drops of fiducial markers on parafilm. After washing 3 times for 10 s in 100 ml beakers filled with Milli-Q water, excessive water was removed with filter paper, and the grids were hold in cross-over forceps and dried on a 30 °C hot plate.

#### Preparation of Tokuyasu samples

HeLa cells were transiently (24 h) transfected with lysosomal associated membrane protein LAMP1-GFP using Effectene as a transfection reagent. After 24 hours in the incubator, the cells were fixed for 15 minutes at room temperature with 4% (w/v) paraformaldehyde (PFA) in 0.1 M phosphate buffer (pH 7.4) added to an equal volume of culture medium, after 15 min replaced with 4% (w/v) PFA in 0.1 M phosphate buffer and further fixed for >2 h. Embedding of cells and preparation of ultra-thin cryo-sections was performed as previously described^19^. Cryo-sections of HeLa cells were collected on TEM grids coated with fiducial markers. Immuno-gold-labeling of the cells transfected with LAMP1-GFP was accomplished using goat anti-GFP (Rockland) and Protein A coupled to 10 nm gold particles (PAG10 nm, Cell Microscopy Core, UMC Utrecht, the Netherlands) as explained by Liou *et al*.^20^. The mitochondrial outer membrane receptor TOM20 of HeLa cells was labelled using primary mouse anti-TOM20 (BD Biosciences, 1:100, 25 µg/ml) and secondary antibody conjugated to Alexa Fluor® 488 (rabbit × mouse, 1:200, 10 µg/ml), followed by PAG10 nm (Cell Microscopy Core, UMC Utrecht, the Netherlands). Finally, the cell nuclei in all samples were counterstained with DAPI.

Derivation and preparation of immortalized FSHD myoblasts/myotubes were previously described elsewhere^21^. Briefly, cells were fixed, embedded, and cryo-sectioned similar to the HeLa cells as described above. Cryo-sectioned cells were collected on TEM grids coated with fiducials, myotubes were stained for actin with Phalloidin-Alexa Fluor® 647 (Cell Signaling Technology, USA), and cell nuclei were counterstained with DAPI. The muscle biopsies from the FSHD patient were obtained from Prof. B. van Engelen, Radboud University Medical Centre in Nijmegen (Netherlands). The experiments on the biopsies were approved by the ethics committee of Radbout University Medical Centre, and the patient provided written informed consent. All experiments were performed in accordance with the guidelines and regulations set out by the ethics committee.

For fluorescence imaging of cryo-sections of HeLa cells and myotubes, TEM grids were sandwiched between 2 cover slides with 50% glycerol in dH_2_O. After fluorescence imaging, sections were rinsed in Milli-Q water, stained with Uranyloxalate pH 7 and embedded in Mehylcellulose/Uranylacetate pH 4 for TEM as described before^22^.

#### Preparation and immuno-labeling of Lowicryl embedded samples

Preparation, fixation, freeze substitution, and Lowicryl HM20 embedding of a muscle biopsy from a FSHD patient and hDUX4 overexpressing C2C12 cells was previously described elsewhere^23,24^. Here, 50 nm sections of Lowicryl HM20 embedded samples were prepared and mounted on TEM grids with fiducials.

Following a previously reported method^23^, sections from FSHD muscle biopsy were blocked using 1% Bovine Serum Albumin (BSA) in PHEM buffer, labelled for Caveolin (Caveolin-specific antibody obtained in Rabbit, BD Transduction laboratories, diluted to 2,5 µg/ml), PAG20 nm (Cell Microscopy Core, UMC Utrecht, the Netherlands), and with Alexa Fluor® 488 (Goat anti-Rabbit, diluted 1:200, Invitrogen). Sections from the C2C12 cells were blocked in the same way, and immuno-labelled for hDUX4 (Mouse anti-DUX4 (P4H2), culture supernatant, diluted 1:100), bridged with a rabbit anti-mouse IgG (1:300, (P4H2), Dako), labelled with PAG10 nm (Cell Microscopy Core, UMC Utrecht, the Netherlands), and with Alexa Fluor® 488 (Goat anti-Rabbit, diluted 1:200 to 10 µg/ml, Invitrogen). The sections were then washed five times two minutes in PHEM buffer and five times two minutes in water, and dried in an open, gridbox, protected from light.

For fluorescence imaging Lowicryl sections of a muscle biopsy of a FSHD patient and C2C12 cells, transfected with hDUX4 were sandwiched between cover slides with Milli-Q water. Lowicryl sections did not undergo any additional preparation for TEM after the fluorescence imaging.

### Imaging

Fluorescence images were acquired with a Deltavision RT widefield microscope using an Olympus 100X-1.40 NA-UPLSApo/O oil objective. This microscope has four detection channels (DAPI, FITC, Texas Red and Cy-5) and is equipped with a Photometrics Cascade II EM-CCD camera. The field of view was 132 × 132 µm^2^ recorded in 1024 × 1024 pixels and the exposure time was 100 ms. Different channels were imaged sequentially and the Deltavision microscope software was used for correcting both chromatic aberrations and stage drift.

Transmission electron microscopy was done on two different TEMs: a Tecnai 12 (Thermo Fischer (FEI), the Netherlands) and a JEM-1010 (JEOL, Japan) both set at 80 kV acceleration voltage. Low magnification TEM images acquired with the TECNAI 12 have pixel sizes of 11.68 nm and image sizes are 24 × 24 µm^2^ while for the JEOL microscope the pixel sizes are 12.82 nm and image sizes are 17.64 × 13.30 µm^2^. High magnification TEM images recorded with the TECNAI 12 have pixel sizes of 0.886 nm and for the JEM-1010 1.64 nm.

### Correlating FM and high magnification TEM images

The correlation method presented here relies on first correlating the FM image with the low magnification TEM image followed by correlating the low magnification TEM image with a high magnification TEM image. Each correlation step is described by a transformation matrix and correlation of the FM and high magnification TEM images is realized by multiplying the transformation matrices of the intermediate correlation steps.

**Figure 1:**
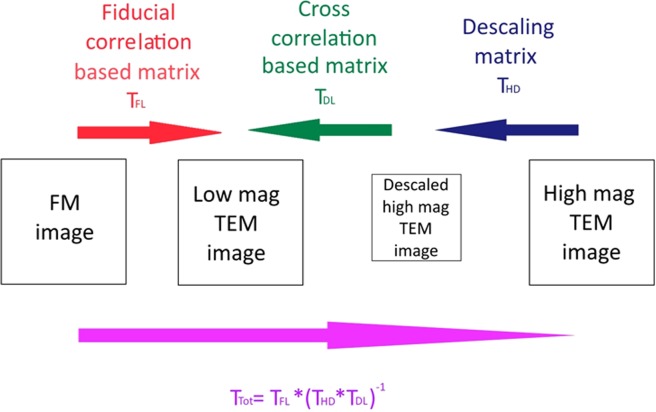
Steps in correlating FM with high magnification TEM images.

Correlation of the FM image and the low magnification TEM images is done on images that differ less than an order of magnitude in size. The center positions of fiducial markers are determined with sub-pixel accuracy in the coordinate systems of the FM and TEM images and used to calculate a transformation matrix. Next, the low magnification TEM image is correlated with a high magnification TEM image. Before this step, the high magnification TEM image is first resampled to match pixel sizes. Since both images show the same morphology and have similar contrast, cross correlation methods can be employed to accurately calculate the transformation matrix for this step. Linear matrices with the following form are used for both transformations:

1 $$T=[\begin{array}{ccc}{S}_{x}\ast \cos \,(\theta ) & {S}_{x}\ast \sin \,(\theta ) & shr\,(x)\\ -{S}_{y}\ast \sin \,(\theta ) & {S}_{y}\ast \cos \,(\theta ) & shr\,(y)\\ tr\,(x) & tr\,(y) & 1\end{array}]$$

here, S_x_ and S_y_ are scaling factors in the x and y direction respectively, *θ* is the rotation angle, shr(x) and shr(y) represent shear and tr(x) and tr(y) translation. The complete procedure to correlate FM and high magnification TEM images is depicted in Fig. 1. A more detailed description of each step is given in the below sections.

#### FM to low magnification TEM

The fluorescence of the fluorescently labelled silica coated gold nanoparticles is very bright and detected in the red (Cy-5) detection channel, well separated from the other fluorescence signals. As a result, they are easy to identify in the FM images. Furthermore, their comparatively large size (120 nm in diameter) makes them stand out in low magnification TEM images. The procedure to correlate FM and low magnification TEM images starts by correcting first for their different orientations and magnifications. This is accomplished by manually picking the centers of fiducials in the FM and TEM images and calculating a transformation matrix using the MATLAB (MathWorks) “fitgeotrans function”. This yields a first, rough transformation matrix; 5 fiducials are sufficient to find this matrix.

Next, a more accurate transformation matrix is determined. In this step all fiducial resembling spots in both the TEM and FM images are automatically detected and fitted with 2D Gaussians using ImageJ (GDSC SMLM plugin)^25^. Here, the low magnification TEM image is first background corrected and the contrast is inverted. The rough transformation matrix is applied on the fitted centers of the fiducials in the FM image and the transformed positions are compared with the positions of fiducials found in the TEM images. If the difference in position is less than 50 pixels (550 nm) they are assumed to correspond with the same fiducial markers. Next, the fitted positions in the FM and low magnification TEM images are used to calculate a precise transformation matrix (T_FL_ in the Fig. 1).

#### Low magnification to high magnification TEM

Cross correlation is used to find the precise transformation matrix between the low and high magnification TEM images. To this end the high magnification TEM image is first resampled to match the pixel size of the low magnification image. The pixel sizes of the low and high magnification TEM images are determined using a calibration sample. The resampling factor, T_HD_, is based on the ratio of the pixel sizes in the high and low magnification images respectively. Next, the resampled image is cross correlated with the corresponding region in the low magnification TEM image. The result of the cross correlation is a transformation matrix T_DL_. This matrix also includes a small correction for the error in the given pixel sizes since pixel sizes slightly vary depending on focusing conditions. Multiplying T_HD_ with T_DL_ yields the complete transformation matrix from high magnification TEM to low magnification TEM.

2 $${T}_{LH}={T}_{HL}^{-1}={({T}_{HD}\ast {T}_{DL})}^{-1}$$

#### FM to high magnification TEM

After finding the precise transformation matrices from FM to low magnification TEM and from high magnification to low magnification TEM, the total transformation from FM to high magnification TEM is found by multiplication of the matrices:

3 $${T}_{Tot}={T}_{FL}\ast {({T}_{HD}\ast {T}_{DL})}^{-1}$$

## Results

### Correlation of FM and high magnification TEM images

**Figure 2:**
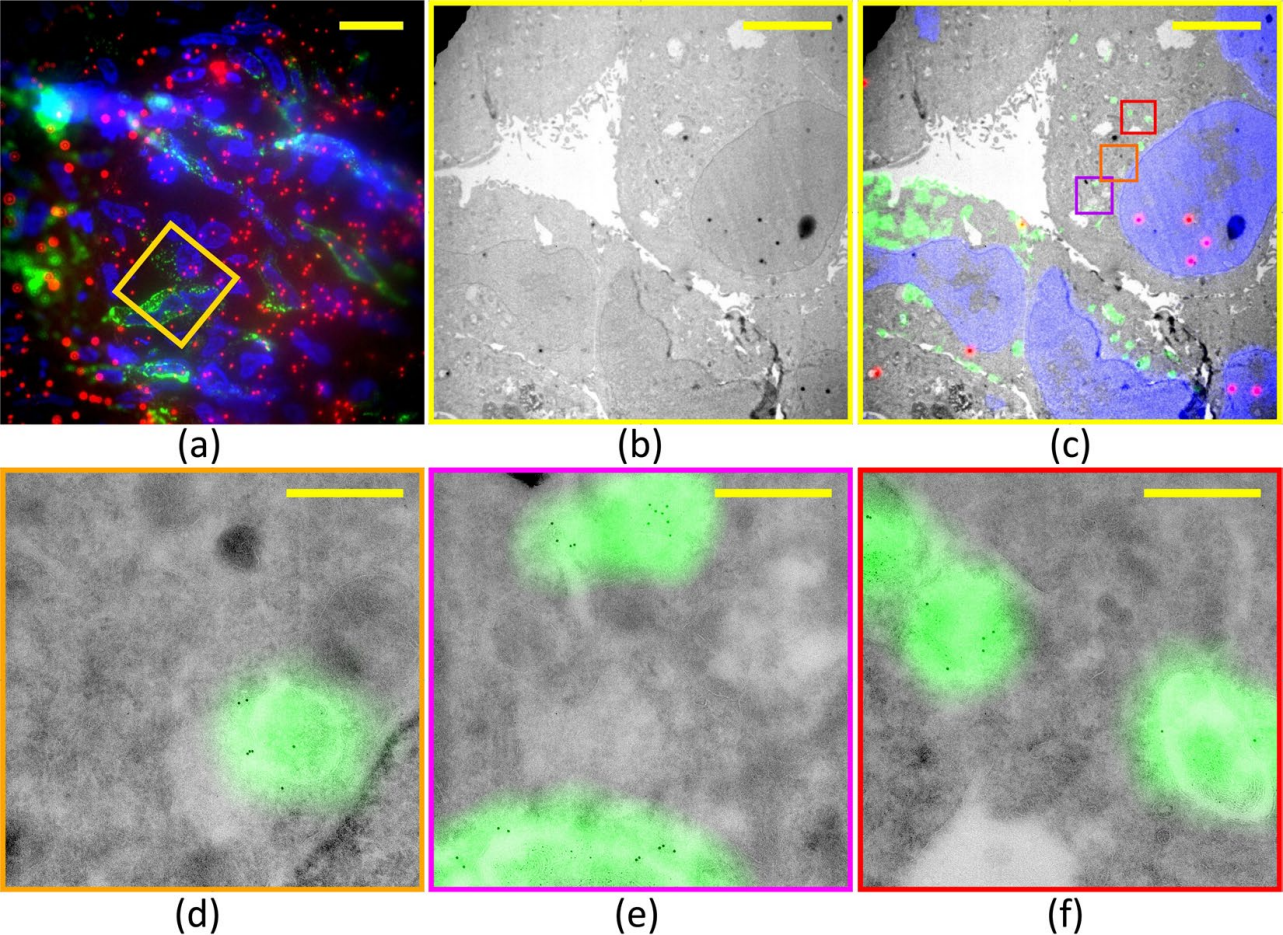
Correlating FM and TEM images of a Tokuyasu section. (**a**) FM image showing nuclei (blue, DAPI), fiducials (red, RITC) and LAMP1-GFP (green). (**b**) Low magnification TEM image of the region indicated by the yellow square in 2-a. (**c**) Overlay of the transformed FM and low magnification TEM images. (**d**–**f**) Overlay of FM and high magnification TEM images for the green, magenta and red squares in 2-c. Excellent agreement is found between the green fluorescence and immuno-gold labeling. Scale bars: 20 µm in (**a**), 5 µm in (**b**–**c**), and 500 nm in (**d**–**f**).

The proposed correlation method was applied on cryo-sections of Hela cells expressing LAMP1-GFP and immuno-gold labelled for GFP on TEM grids decorated with fiducial markers. The nuclei of the cells were stained using DAPI. The fluorescence of DAPI, LAMP1 -GFP and the fiducials was detected sequentially and represented in blue, green and red respectively in Fig. 2a. The fiducial markers are clearly visible as red spots in the image. In Fig. 2b the low magnification TEM image of the area indicated by the yellow square in Fig. 2a is shown. Figure 2c shows the overlay of the FM image and the low magnification TEM image. In this figure three ROIs are marked by the orange, magenta and red squares. High magnification TEM images overlaid with FM images of these ROIs are given in Fig. 2d–f. There is good agreement between the presence of the gold particles and the green fluorescence, in particular in Fig. 2d,e.

Note that there are no fiducial markers present in the high magnification TEM images in Fig. 2d–f. Existing fiducial based methods would require 6–10 fiducial markers to be present in the field of view^8,9,26^.

### Correlation accuracy

#### Accuracy of correlating FM and low magnification TEM images

**Figure 3:**
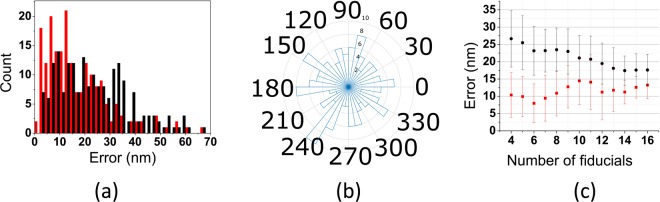
Accuracy analysis of transformation from FM to low magnification TEM images using two different approaches (see text). (**a**) Frequency plot of the error. (**b**) Frequency plot of direction of error. (**c**) Mean error vs number of fiducials used for correlation.

A direct measure for the accuracy of the correlation is the difference between the actual and transformed positions of the fiducial markers. Here, the (accurate) transformation matrix between the FM and low magnification TEM image was used to transform the fitted positions of the fiducials in the FM image to positions in the low magnification TEM image. Next, the difference between these positions and the actual positions of the fiducial markers in the low magnification TEM image was calculated. This procedure was repeated for all fiducials in 17 FM/low magnification TEM images (image size is 24 × 24 µm^2^) and resulted in the error distribution in Fig. 3a (red bars). It is based on a total of 194 fiducials, has a mean value of 17 nm and a median value of 13.5 nm. This error analyses also yields information about the direction of the errors. The polar histogram of errors is shown in Fig. 3b and reveals an isotropic distribution. Figure 3c (red squares) depicts the dependency of the errors on the number of fiducial markers used to calculate the transformation matrix. Here, the 17 analyzed images contain between 4 to 18 fiducials per image, all subsets with at least 4 fiducial markers were used in the analyses.

Another approach we followed to assess the accuracy was as is described by Kukulski *et al*.^8^. For an image containing N fiducials, transformation matrices are calculated for all possible sets of N-1 fiducials. For each set the expected position of the left-out fiducial is calculated using the transformation matrix associated with the N-1 remaining particles. The difference between the calculated and the real position of this fiducial can now be used as a measure for the error in the position. Here, this procedure was applied to all fiducials in a number of 24 × 24 µm^2^ images to obtain an error distribution, Fig. 3a black bars (mean value 23.8 nm and median value 22.0 nm). Figure 3c shows the dependency of the error on the number of used fiducial markers.

The fundamental difference between the two approaches to estimate the error is that the first approach represents the correlation accuracy of the fiducials used in finding the transformation matrix while the latter depicts the correlation accuracy of fiducials not used for finding the transformation matrix. The transformation matrix will produce zero overlay error in the first approach if less than 4 fiducials are used (the transformation matrix has 7 degrees of freedom) and for larger numbers of fiducial the error rises. The second approach results in the largest error for low numbers of fiducials; removing one fiducial from the analysis results in comparatively large changes in the error. Both approaches will converge to approximately the same error value (~15 nm) if sufficient numbers of fiducials are used. The advantage of the first approach is that the accuracy is a by-product of the matrix analysis while the second approach requires an extra analysis to determine the overlay accuracy.

We note that the error analyses sometimes revealed fiducials with high errors, more than twice the median error. Upon closer analyses these outliers often were sitting on folds in the section or other unfavorable places. These outliers were removed from the error analyses and also not used for calculation of the transformation matrices.

#### Accuracy of correlating low and high magnification TEM images

**Figure 4:**
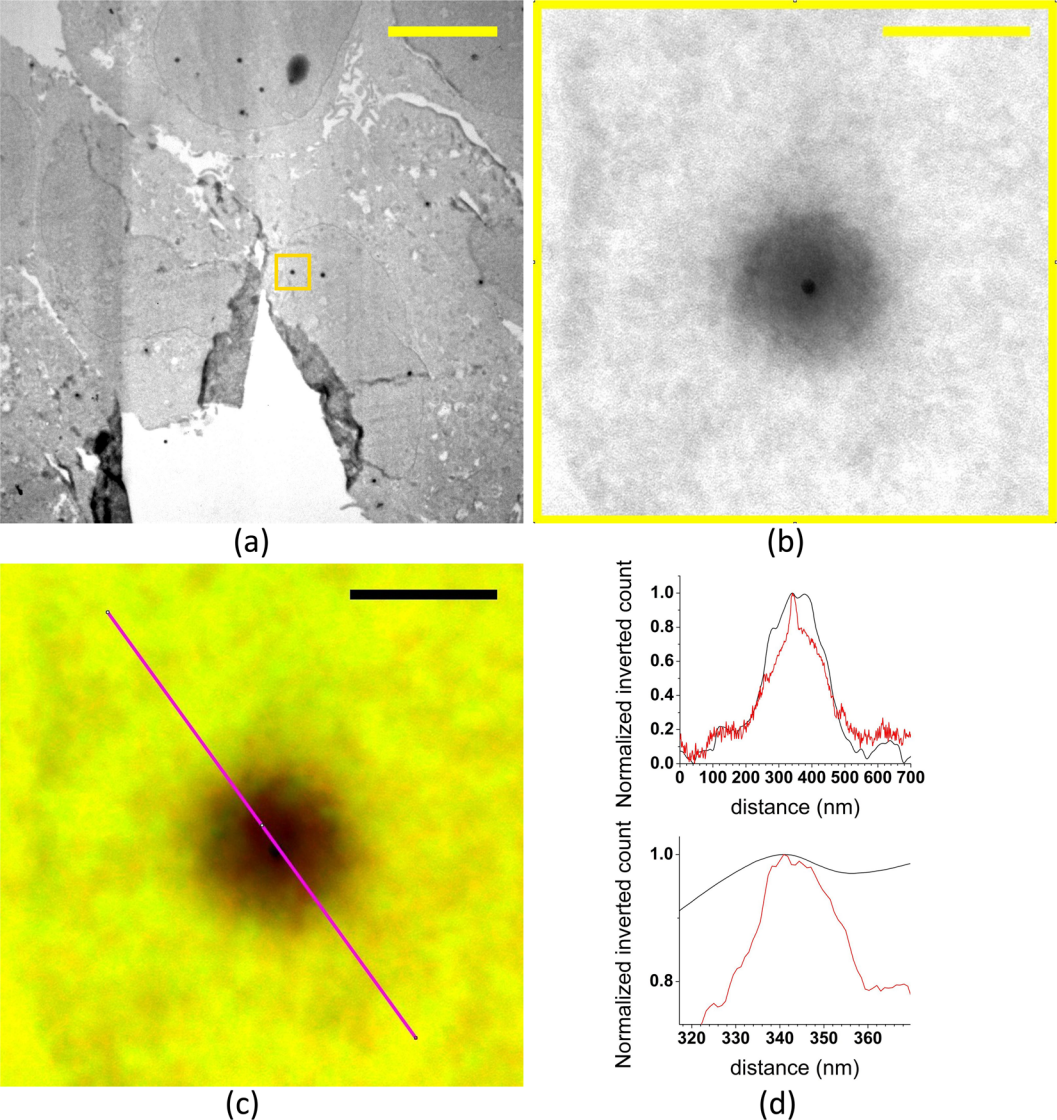
Accuracy of cross-correlating low and high magnification TEM images of fiducial markers. (**a**) Original low magnification TEM image, the box indicates the position of the high magnification image. (**b**) Original high magnification TEM image. (**c**) Overlay of the high magnification and the transformed low magnification TEM images. (**d**) Cross section through the fiducial marker along with magenta line in c) as well as a closer look at the top peak of the profile. Red plot is for original high magnification TEM and black is for transformed low magnification TEM. Scale bars: 5 µm in (**a**), 200 nm in (**b**) and (**c**).

Correlating the low and high magnification TEM images is accomplished by cross-correlation. To measure the accuracy of this step 21 low and high magnification images of the same fiducials were used. The correlation error is found by plotting a line profile through the center of a fiducial in low and high magnification and comparing the central peaks for both cases. An example is shown in Fig. 4: (a) shows the original low magnification TEM image, the box indicates the area of the high magnification image, (b) - the original high magnification TEM image, (c) - the overlay of the transformed low magnification TEM and high magnification TEM images. The magenta line shows the place where a line profile is plotted through both transformed low magnification and original high magnification TEM images. The overlap between the fiducial markers in transformed low and high magnification images is near perfect. The line profiles through the center, Fig. 4d, confirm this. The mean value of the position error of particles was found to be 5.4 nm (median value is 4.1 nm). A high accuracy is indeed expected from cross correlating images recorded at different magnifications.

#### Accuracy of correlating FM and high magnification TEM images

To assess the overlay accuracy of the FM and high magnification TEM images the complete correlation procedure was carried out on FM and high magnification TEM images of the same area containing fiducial markers (23 fiducials measured in high mag TEM). By directly comparing the centers of the positions of the fiducial markers in FM and TEM images a mean correlation accuracy of about 27 ± 11 nm was found (N = 23, Fig. 1 in supporting information(SI)). This accuracy is in agreement with the previously estimated errors based on much better statistics ($\sqrt{{17}^{2}+{5.4}^{2}}=17.8\,{\rm{nm}}$; 17 nm for FM to low magnification TEM and 5.4 nm for Low magnification TEM to high magnification TEM). The total correlation error is dominated by the FM to low magnification TEM transformation step.

### Dependency of the method on setup and sample type

To investigate the suitability of the method for use with different TEMs and different types of specimens, experiments were carried out on both Tokuyasu and Lowicryl embedded specimens using two different electron microscopes, a Tecnai 12 and a JEM-1010.

**Figure 5:**
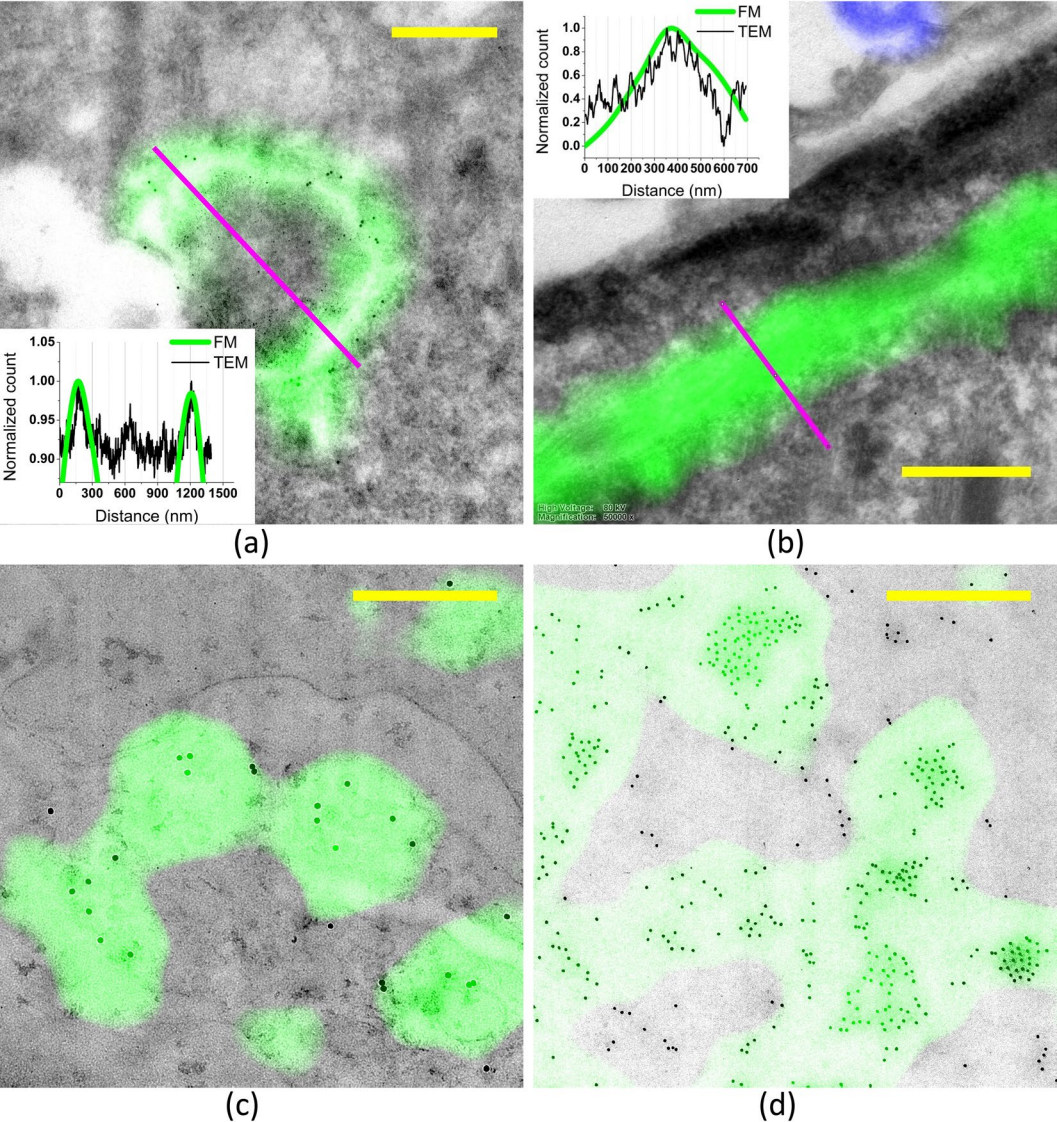
Overlays of FM and high magnification TEM images: (**a**) Tokuyasu section of HeLa cells transiently transfected with LAMP1-GFP and LAMP1 immuno-gold labelled, (**b**) Tokuyasu section of immortalized FSHD myoblasts/myotubes stained for actin with Phalloidin-Alexa Fluor® 647, (**c**) Lowicryl section of muscle biopsy of a FSHD patient, labelled for Caveolin both with PAG20 nm and Alexa Fluor® 488 and (**d**) Lowicryl section of C2C12 cells transiently transfected with hDUX4 and immuno-labelled for hDUX4, labelled with both PAG10 nm and Alexa Fluor® 488. (**a**,**c**,**d**) are measured with a Tecnai 12 and (**b**) is measured with a JEM-1010. The scale bar in all of these images is 500 nm.

Figure 5a,b show the overlays of correlated FM (green) and high magnification TEM images (black and white) of 2 different Tokuyasu samples. In Fig. 5a, a section of a HeLa cell transiently transfected with LAMP1-GFP and labelled with anti-GFP and PAG10 nm is shown. The TEM image was acquired using the TECNAI 12. At the location of the green fluorescence signal in Fig. 5a also gold particles representing LAMP1 are visible, confirming that both labels are targeted to lysosomes. Figure 5b shows Tokuyasu cryo-sections of immortalized FSHD myoblasts/myotubes stained for actin with Phalloidin-Alexa Fluor® 647. Here, the TEM image was recorded using the JEM-1010.

The insets in Fig. 5a,b show profiles corresponding to the magenta lines in the images. From comparing the profiles of the fluorescence signal (green) and the (contrast-inverted) high magnification TEM signal (b/w), the error in the overlays can be estimated. In Fig. 5a, the maxima of the FM and TEM peaks are separated by only 5 nm and in Fig. 5b by about 14 nm. This excellent correlation is significantly better than the previously found mean accuracy of ~17.8 nm and is at the tail of the error distribution in Fig. 3a.

Figure 5c,d show the overlays of FM and TEM images of Lowicryl sections recorded using the Tecnai12. Figure 5c is a section from a muscle biopsy of a FSHD patient. It shows endothelial cells of a capillary immuno-labelled for Caveolin both with PAG20 nm and with Alexa Fluor® 488 and d) is a section of C2C12 cells, transfected with hDUX4 and immuno-labelled for hDUX4, both with PAG10 nm and with Alexa Fluor® 488. Figure 5d shows a part of a nucleus overexpressing hDUX4. All images show excellent correlation between the fluorescence signal (green) and regions with a high density of the gold label.

Finally, we note that in none of these examples fiducial markers are present in the field-of-view. As a result, no parts of the specimen are shadowed by fiducials. Nevertheless, the correlation accuracy is high.

## Discussion

In this work high accuracy correlation of fluorescence and TEM images is realized in three steps. First, FM images are correlated with low magnification TEM images. For this first correlation, a low number of fiducials markers based on a gold core and a fluorescently labelled silica shell are used. These fiducials have good visibility in both FM and low magnification TEM. Next, the low magnification TEM images are correlated with high magnification TEM images. The correlation of these images is performed using a cross correlation procedure and is free from any user intervention. The final step consists of multiplying the transformation matrices obtained in the first two steps. This yields the matrix that describes the coordinate transformation going from the FM to the high magnification TEM image.

The accuracy of the FM to low magnification TEM mapping is very high. When 10 or more fiducial markers are used in the FM/low magnification TEM images the accuracy is around 15 nm. In view of shrinkage effects of sections observed after exposure to the electron beam the high accuracy may be somewhat surprising. However, in our method the sections are first imaged in FM, which does not introduce distortion of the specimen, and next in TEM at low magnification. At low magnifications electron doses are low and only minor distortions of the sample are expected.

Shrinkage of the ultra-thin sections due to exposure to the electron beam can occur both in depth and in plane of the sections. In plane shrinkage depends on thickness, type of embedding and the type of biological material. Due to e.g. variations in thickness shrinkage can be non-linear. Importantly, shrinkage strongly depends on electron dose and can be particularly strong for high magnification EM imaging. Values of up to 30% have been reported^27–29^, which can result in large correlation uncertainties.

The influence of shrinkage due to exposure to the electron beam on the correlation accuracy was investigated. First, a low magnification TEM reference image of 24 × 24 μm^2^ was recorded of a Lowicryl section. Next, the same 24 × 24 μm^2^ area was imaged using high magnification TEM in order to expose it to a high electron dose. This was accomplished by sequentially imaging small, 1.8 × 1.8 μm^2^, parts of the larger image. Finally, another low magnification image was recorded of the same area and positions. Shrinkage was observed and quantified by the difference in position of fiducial markers in the image.

Figure 2 in the SI shows the overlay of the fluorescence and the first low magnification TEM images. Little or no shrinkage is visible confirming that the low magnification TEM imaging results in no significant shrinkage. High magnification TEM imaging of the sample, however, deteriorates the overlays accuracy, most probably due to shrinkage. This result stresses the need to acquire the low magnification TEM image before the high magnification TEM image. Figure 3 in the SI is the quantitative analysis of the error distribution before and after shrinkage of the sample. The other important message is that skipping the low magnification imaging in favor of using a mosaic stitching strategy to create a large field of view can decrease the accuracy of the correlation due to different degrees of local shrinkage of the sample. Therefore, the best strategy to obtain a high correlation accuracy is to first record the low magnification TEM image. After this initial step high magnification TEM images can be recorded.

The accuracy of the low magnification TEM to high magnification TEM transformation is better than 5.4 nm. This high accuracy is the result of cross correlating images with the same features and contrast. The raw images only differ in magnification (pixel size) and after rescaling the high magnification image about 10^4^ points are correlated. Local shrinkage apparently has little effect, only a very small part of the sample is imaged, and shrinkage is taken into account by correlating the large number of pixels.

We noticed that by using a linear transformation matrix there is no need for a homogenous distribution of fiducial markers. Even when the fiducials are predominantly located at one side of the image a linear transformation yields accurate overlays at the fiducial-free side of the image. Results from literature indicate that this is not the case when non-linear transformations are used^27,30–32^. Here, a more or less homogenous distribution of fiducials is required for high overlay accuracy over the whole field of view.

The overall accuracy of the correlation procedure is dominated by the accuracy of the FM to low magnification TEM step. This accuracy depends on the number of fiducial markers available for correlation. In practice we have used 10 or more fiducial markers in our correlative imaging experiments, which results in a mean overall correlation error of about 18 nm. The spread in the correlation accuracy is, however, comparatively high (~50%, see Fig. 3). This is due to several factors. Specimen preparation for TEM after FM imaging and transporting the specimen between the FM and TEM may introduce deformations. The sample may also shrink due to exposure to the electron beam. In addition, there is a small uncertainty in localizing the center of fiducials in the FM and low magnification EM images. The distribution of fiducials through the sample affects the correlation accuracy of features. Features close to a number of fiducials will have higher correlation accuracy. In some cases, extremely high accuracies are found, ~5 nm in Fig. 5a. This is likely due to low local distortion of the specimen in combination with fiducial markers lying nearby the ROI. In practice we found the correlation errors to be in the range 5–30 nm.

The method proposed in this paper has the potential to be extended to 3D CLEM methods. However, depending on the type of 3-D EM method different challenges have to be overcome. In, for instance, correlative FIB-SEM and confocal microscopy measurements, the optical resolution in the depth direction is in general poor and optical distortions usually increase with depth. This complicates the correlation process and impairs accuracy. Other combinations of imaging modalities have their own challenges but in general the method proposed here should work. A common issue with most techniques is that for 3D CLEM fiducials need to be more or less homogeneously distributed throughout the volume studied. This imposes constrains on sample preparation.

At present the whole procedure except for the first manual selection of fiducials for the first coarse transformation is automated. This includes the rejection of outlier fiducials and finding all transformations. The automated part was carried out on a regular PC (Intel I5 processor, 16 Gb memory) and the total execution time for correlating FM and high magnification TEM images was less than 2 min. Finally, we note that there are prospects to fully automate the correlation procedure.

## Conclusions

Here we report a method to accurately correlate FM and high magnification TEM images. The method is simple, robust and has an accuracy around 5–30 nm. It is important to prevent shrinkage of the sample. Here, this is accomplished by recording the low magnification TEM image before the high magnification image. The use of cross correlation to correlate low and high magnification TEM images adds to the speed of the analyses and does not significantly contribute to the total correlation error. Importantly, the cross-correlation step is feature based and does not require fiducial markers or gold particles in the field of view.

We envisage that the correlation method can also be employed to correlate super resolution FM and high magnification TEM images. This would likely result in even lower correlation errors and offer the prospect of accurately mapping of fluorescent markers in TEM images for which no TEM immuno-labeling alternatives are present.

## Supplementary information

Supporting information
