# Supplementary material for: High accuracy, fiducial marker-based image registration of correlative microscopy images
Source: Sci Rep. 2019 Mar 1;9:3211. doi: 10.1038/s41598-019-40098-4 (PMC6397213; doi:10.1038/s41598-019-40098-4)
Supplement: Supplementary file 1 — Supporting information [file 41598_2019_40098_MOESM1_ESM.pdf]

High accuracy, fiducial marker-based image registration of correlative microscopy images.

Sajjad Mohammadian, Jantina Fokkema, Alexandra V Agronskaia, Nalan Liv, Cecilia de Heus, Elly van Donselaar, Gerhard A. Blab, Judith Klumperman and Hans C Gerritsen

S1 Correlation accuracy of FM and high magnification TEM images

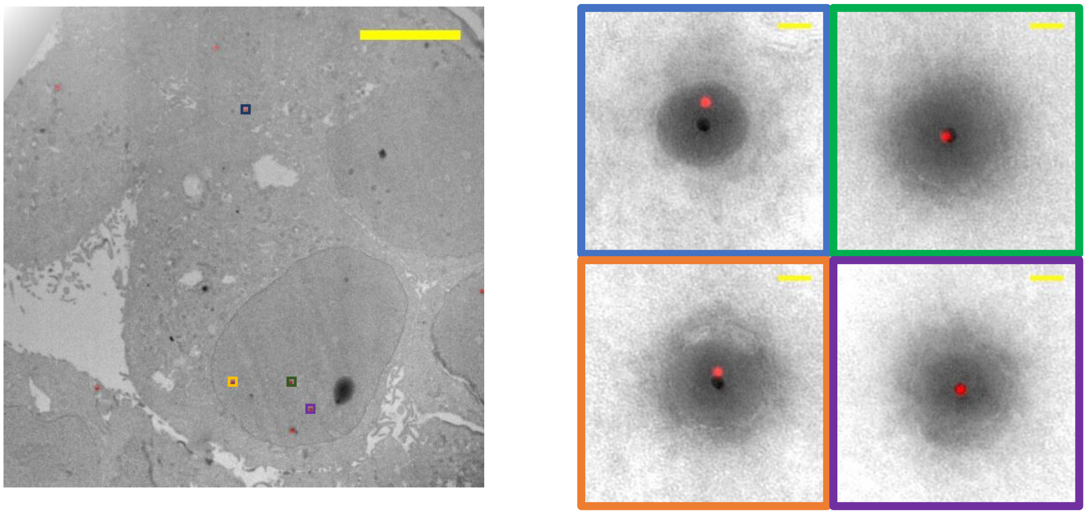

(a) (b)

Figure 1. Overlay accuracy of the FM and high magnification TEM images. (a) The red spots indicate the centers of the fiducial markers in fluorescence overlaid with the low magnification TEM image. (b) High magnification TEM images of the areas indicated by the colored boxes (blue, green, orange and magenta) in (a), overlaid with the centers of the fiducial markers in fluorescence. scalebar: 5 µm in (a) and 50 nm in (b).

Figure 1 shows how the correlation accuracy of FM and high magnification TEM images was determined using the centers of fiducial markers. The location of the fiducials in fluorescence is found from fitting with a Gaussian function. These locations were then transformed using the transformation matrices and mapped onto the TEM images (red dots) using the transformation matrices. Figure 1a shows the overlay of a number of fiducials in FM and low magnification TEM and Figure 1b the overlay of these fiducials in FM and high magnification TEM. The correlation accuracy for a specific fiducial marker was found by calculating the difference between the fitted center of the FM image and the fitted center of the gold core of the fiducial in high magnification TEM.

S2 Effect of shrinkage on overlay accuracy

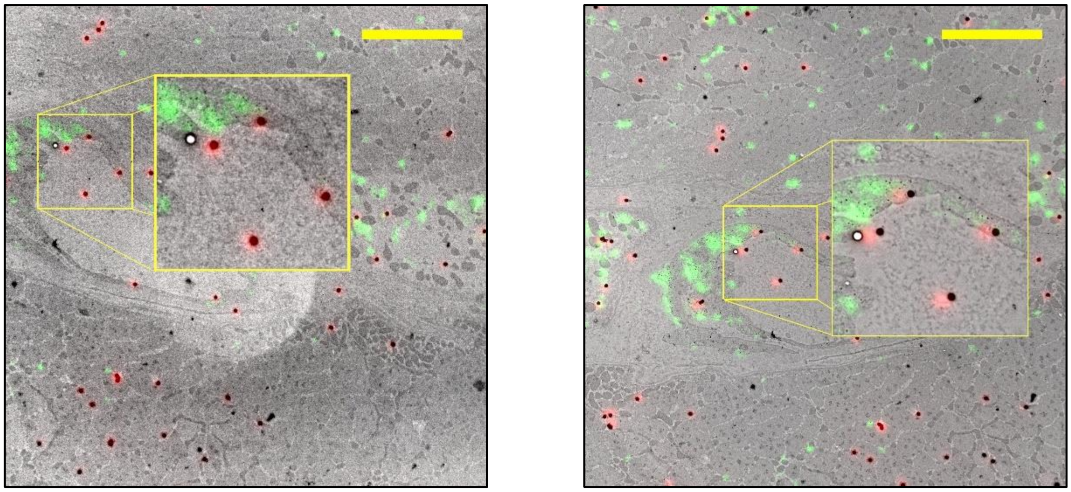

(a) (b)

Figure 2. Effect of specimen shrinkage due to exposure to the electron beam on correlation. (a) Overlay of FM and low magnification TEM images before exposing the specimen to a high electron dose. (b) Overlay of FM and low magnification TEM images after exposure to a high electron dose. scalebar: 5 µm

Figure 2 shows the effect of exposure to the electron beam on the overlay of FM and TEM images. In (a) the overlay of a FM and low magnification TEM image is shown. The specimen was not exposed to an additional electron dose. The positions of the fiducial markers in FM and low magnification TEM images correlate very well. In (b) a similar overlay as in (a) is shown. Here, the specimen was exposed to an additional high electron dose before recording the low magnification TEM image. Now, a clear offset is observed between the fiducials in the FM image from a) and the low magnification TEM image. The offset is attributed to shrinkage due to exposure to the electron beam.

S3 Quantification of the correlation error due to shrinkage

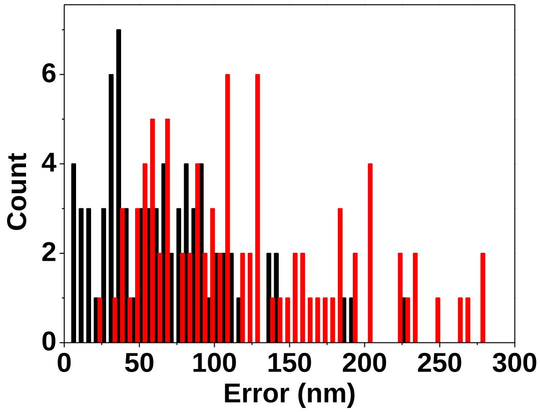

Figure 3. Correlation error distribution of fiducial markers in FM and low magnification TEM images, with shrinkage (red bars) and without shrinkage (black bars).

The error in the correlation due to shrinkage after exposing the specimen to a high electron dose was investigated. FM and low magnification TEM images were recorded and the correlation error in the positions of 88 fiducials was determined (Figure 3, black distribution). Next, the same areas in the specimen were exposed to a high electron dose and the correlation error in the positions of the same fiducials with respect to the original FM image was again determined (Figure 3, red distribution). Comparison of the distributions show that there is a strong effect of specimen shrinkage on the correlation accuracy (shift~ 60 nm).
